# Protective effects of ischemic postconditioning on skeletal muscle following crush syndrome in the rat

**DOI:** 10.1590/ACB360701

**Published:** 2021-09-03

**Authors:** Wei Wang, Yuan Wang, Jing Yang

**Affiliations:** 1 MD. Department of Orthopaedics - First Medical Center of PLA General Hospital - Beijing, China.; 2 MS. Department of Orthopaedics - First Medical Center of PLA General Hospital - Beijing, China.; 3 MD. Department of Pain - First Medical Center of PLA General Hospital - Beijing, China.

**Keywords:** Crush Syndrome, Reperfusion injury, Ischemic Postconditioning, Rats

## Abstract

**Purpose:**

To investigate the effect of ischemic postconditioning (IPostC) on skeletal muscle and its optimal protocol.

**Methods:**

This article is about an animal study of rat model of crush syndrome. Sixty rats were randomized into nine different IPostC intervention groups and a control group. The anesthetized rats were subjected to unilateral hindlimb 3-kg compression with a compression device for 6 h, followed by nine different IPostC intervention protocols.

**Results:**

Serum levels of creatine kinase (CK) at 3 h post-crush became 2.3-3.9 times among all 10 groups after crush. At 72 h post-crush, serum CK level was reduced to 0.28-0.53 time in all intervention groups. The creatinine (CREA) level in the control group was elevated to 3.11 times at 3 h post-crush and reduced to1.77 time at 72 h post-crush. The potassium (K+) level in the control group was elevated to 1.65 and 1.41 time at 3 and 72 h post-crush, respectively.

**Conclusions:**

Our IPostC intervention protocols can effectively protect rats from crush-induced elevation of serum CK, CREA, and K+ levels. The timing of IPostC intervention should be as early as possible, to ensure the protective effect.

## Introduction

Crush injury is defined as a compression of the extremities or other parts of the body, causing muscle swelling and/or neurological disturbances in the affected areas, which commonly occurs in earthquakes, vehicular crashes, and industrial, mining and farming accidents^1^. Severe crush injury may further progress to a life-threatening condition, namely crush syndrome, which is characterized by systemic symptoms, such as acute kidney injury, hypotension, hypovolemic shock, and hyperkalemia^2^. Crush syndrome is due to rhabdomyolysis and ischemia-reperfusion (I/R) injury^3^. I/R injury is a pathological condition caused by blood return (reperfusion) to an ischemic tissue^4^, which can induce a cascade of acute inflammatory events, leading to cell death and tissue necrosis and dysfunction^5^. I/R injury has been extensively investigated in the vital organs, such as heart, kidney, liver, brain, and intestine^6^^–^ 10. Nevertheless, I/R injury to skeletal muscles can also cause severe systemic problems, such as crush syndrome^11^.

Ischemic postconditioning (IPostC) refers to repeated short cycles of ischemia and reperfusion before the sustained reperfusion^12^, and its protective effects on skeletal muscle have been demonstrated in both human and animal skeletal muscle models^13^^–^ 17. However, contradicting these findings, Mansour *et al*.^18^ have reported that IPostC aggravates skeletal muscle injury in a rat model of I/R injury. Lintz et al.^19^ showed that IPostC had no protective effect in a rat model of I/R injury. Lintz et al.^20^ also reported that IPostC had neither protective effect on skeletal muscle injury nor avoided apoptosis induction in rats submitted to partial ischemia and reperfusion. These findings suggest that the effect of IPostC on I/R injury on skeletal muscle remains to be further investigated.

Therefore, this study aimed to investigate the effect of IPostC on skeletal muscle I/R injury and the optimal protocol of IPostC.

## Methods

### Establishing the rat model of crush injury

This study was conducted in the Orthopedic Laboratory of Chinese PLA General Hospital, Institute of Orthopaedic Research at Chinese PLA General Hospital, Medical School of Chinese PLA, Department of Orthopaedics, First Medical Center of PLA General Hospital. All protocols used in this study were approved by the Institutional Animal Care and Use Committees (IACUCs) of our hospital.

**Figure 1:**
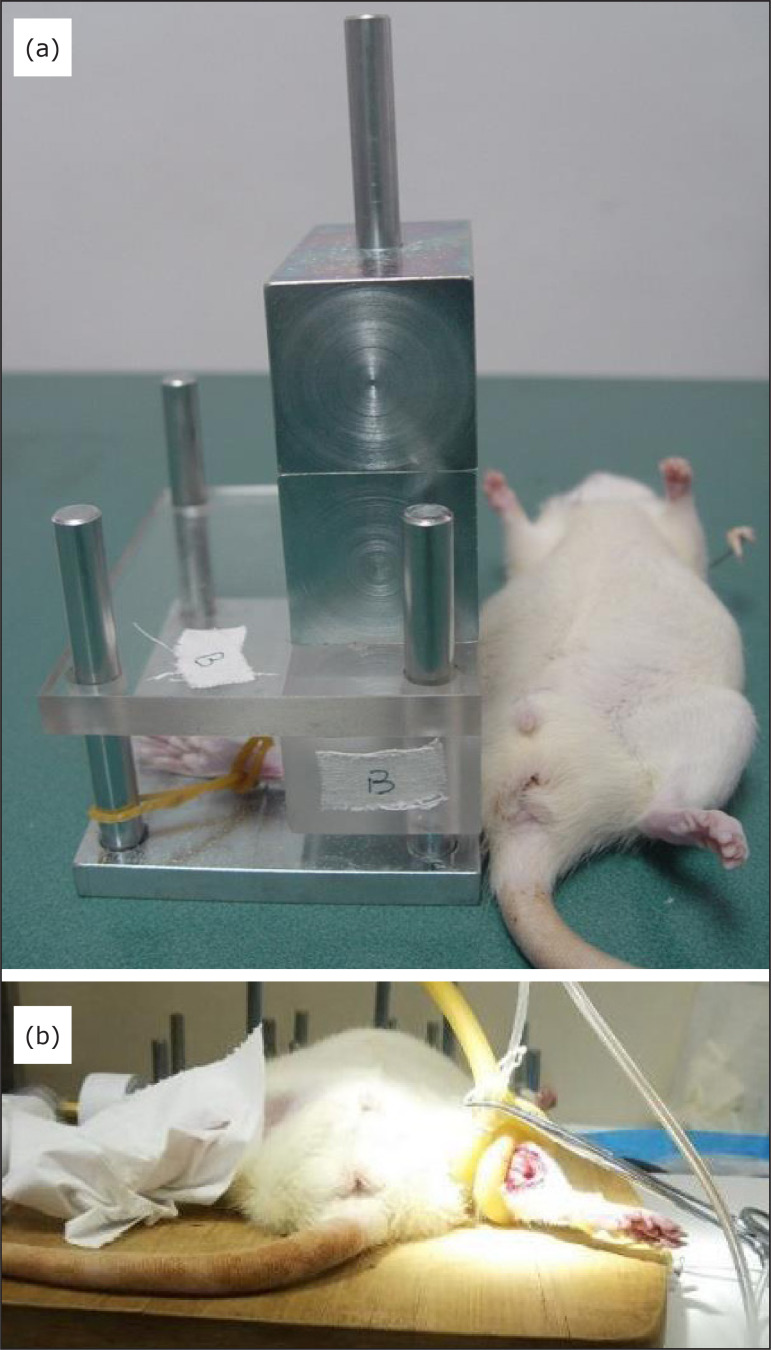
The homemade devices for compression and ischemic postconditioning (IPostC). (**a**) The device for compression of rat hindlimb, which was made based on a similar device designed by Asai Works Co. (Osaka, Japan). (**b**) A homemade tourniquet with a pressure sensor for loosening or clamping the rat crushed hindlimb for IPostC.

The rat model of crush injury was established as previously described^21^. The device for compression of rat hindlimb (Fig. 1a) was produced based on a similar device designed by Asai Works Co. (Osaka, Japan)^22^. Briefly, the rat was anesthetized with 10% chloral hydrate (40 mg/kg), lying on its backs on a warm blanket, and the hindlimbs were fixed with a rubber band on the metal platform of the crush injury device with a 3-kg-weight metal for 6 h (Fig. 1).

During the experiment, when the rat head was struggling, 10% chloral hydrate (4 mg/kg) was injected to maintain the anesthesia state.

### Ischemic postconditioning

**Figure 2:**
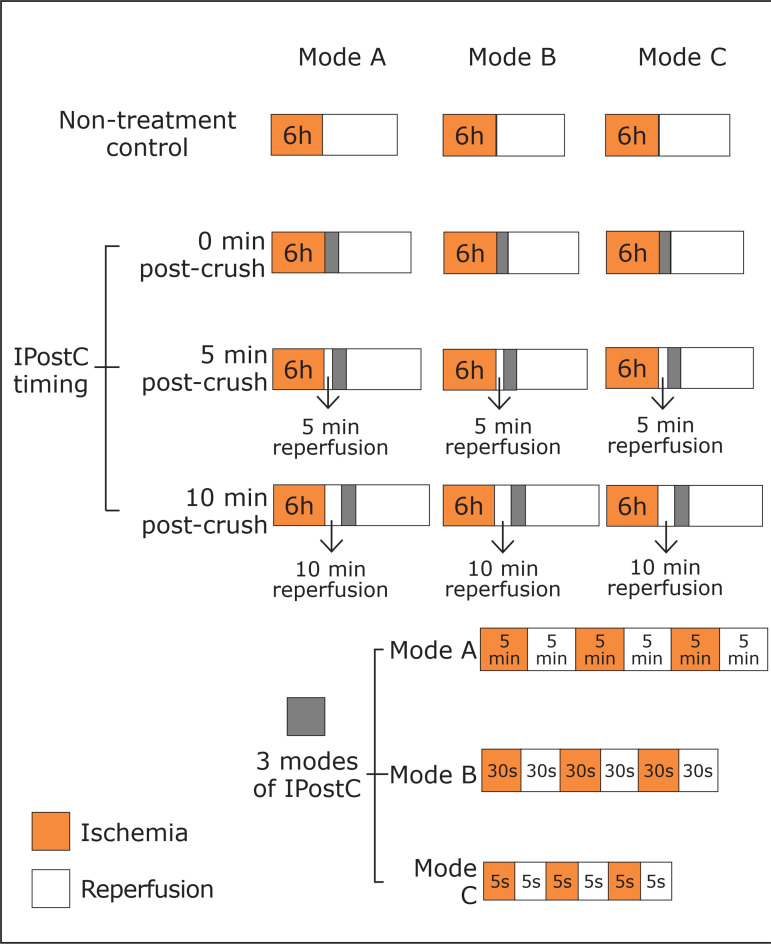
Protocols of ischemic postconditioning (IPostC) of the nine IPostC groups. There were three timing of IPostC intervention post-crush: immediate (0 min post-crush), 5 min post-crush, and 10 min post-crush. There were three modes of IPostC: mode A IPostC was performed by three 5-min cycles of reperfusion and 5-min of reocclusion by tourniquet; mode B IPostC was performed by three 30-sec cycles of reperfusion and 30-sec of reocclusion by tourniquet; mode C IPostC was performed by three 10-sec cycles of reperfusion and 10-sec of reocclusion by tourniquet. Therefore, a total of nine IPostC intervention groups was set up (n = 6 for each group).

IPostC was performed by using a homemade tourniquet with a pressure sensor for loosening or clamping the rat crushed hindlimb (Fig. 1b). There were three timing of IPostC intervention post-crush: immediate (0 min post-crush), 5 min post-crush, and 10 min post-crush (Fig. 2). There were three modes of IPostC (Fig. 2). Mode A IPostC was performed by three 5-min cycles of reperfusion and 5-min of reocclusion by tourniquet. Mode B IPostC was performed by three 30-sec cycles of reperfusion and 30-sec of reocclusion. Mode C IPostC was performed by three 10-sec cycles of reperfusion and 10-sec of reocclusion. Therefore, a total of nine IPostC intervention groups was set up: 0A, 0B, 0C, 5A, 5B, 5C, 10A, 10B, and 10C.

### Data collection

All rats measured the circumference of the compressed hindlimb at the proximal end (compression center) before crush and 3 h post-crush. A sampling tube was fixed between the two feces of all rats to collect urine, in order to observe the occurrence of hematuria from the beginning of compression to 3 h post-crush.

A 1.5-mL arterial blood sample was drawn from all rats before crush (baseline value), 3 h post-crush and 72 h post-crush to determine biochemical indicators (aspartate aminotransferase [AST], alanine aminotransferase [ALT], serum potassium [K^+^], creatinine [CREA], creatine kinase [CK], and blood urea nitrogen), while the same amount of saline was injected into rat via the jugular vein.

For histopathological analysis, the rat was sacrificed under deep anesthesia at 24 h post-crush, and the compressed muscles tissue sample was collected for pathological section and hematoxylin and eosin (H,E) staining.

### Statistical analysis

Continuous data were indicated with mean ± standard deviation (SD), while categorical data were indicated with number and percentage (%). For comparisons of means among all groups, one-way analysis of variance (ANOVA) was used, and Dunnett’s test as post-hoc comparisons (control group as reference). To further investigate the association between independent variables and outcome index, a multivariate linear regression under generalized estimating equation (GEE) model was used.

All rat’s results were measured at three time-points: before crush, 3 h post-crush, and 72 h post-crush. An AR(1) correlation matrix was adopted for the repeated measure data. A p < 0.05 would be recognized as reaching the significance of each test, two-tailed. All analyses were performed using IBM SPSS Version 25 (SPSS Statistical Crush syndrome V25, IBM Corporation, Somers, NY, United States).

## Results

### The study design and basic information of rats

A total of 61 rats was included in this study, six for the control group, and 54 for different IPostC intervention groups. There were three timing of IPostC intervention post-crush: immediate (0 min post-crush), 5 min post-crush, and 10 min post-crush; and three modes of IPostC were adopted: A, B, and C. Therefore, a total of nine groups crossed from IPost timing and types were included, six rats for each group. The outcome measurement of each rat was recorded at three time-points: before crush, 3 h post-crush, and 72 h post-crush.

The bodyweight of rats was significantly reduced after crush damage (before and post-crush: 384.90 ± 60.15 g *vs*. 354.89 ± 62.27 g, p < 0.001). The leg circumference before and post-crush were 7.75 ± 0.46 and 8.46 ± 0.54 mm, respectively (p < 0.001). No rats occurred hematuria during the study.

### Histopathological analysis

**Figure 3:**
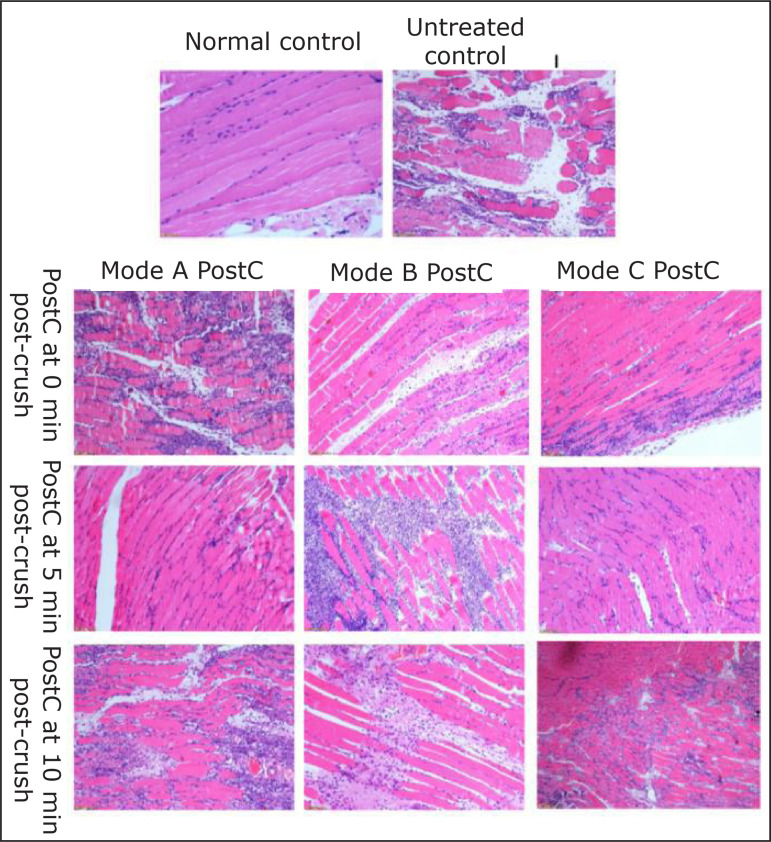
The histopathological analysis of the compressed muscles tissue sample. The rat was sacrificed at 24 h post-crush, and the compressed muscles tissue sample was collected for hematoxylin and eosin staining. The representative images of the normal control, non-treatment control, and nine ischemic postconditioning intervention groups were shown.

To evaluate the protective effect of IPostC, the compressed muscles tissue sample was collected at 24 h post-crush for H,E staining (Fig. 3). In the normal group, the regular striated muscle structure could be observed, and the muscle fibers were arranged in an orderly manner, with intact structure. However, in the untreated control, there were only a few residual muscle fibers, a large amount of inflammatory cell infiltration and piecemeal necrosis. The striated structure of muscle fibers disappeared, and the arrangement was disordered or broken (Fig. 3), suggesting that the model of crush injury was successfully established.

In the 0A, 5B, and 10A, 10B and 10C group, there were a lot of structural damage and necrosis in the muscle fibers and infiltration of inflammatory cells (Fig. 3), similar to the untreated control group, but the severity was attenuated varying degrees.

In turn, in the 0B, 0C, 5A and 5C groups, most of the muscle fibers were arranged in an orderly manner, with intact structure and some local infiltration of inflammatory cells (Fig. 3).

### Comparison of seven indexes at three timepoints

The outcome measurements of seven indexes (ALT, AST, calcium ion concentration – Ca^2+^, CK, CREA, K^+^, and UREA) before crush, 3 h post-crush, and 72 h post-crush were summarized in Tables 1, 2, and 3, respectively. Before crush, only ALT level was significantly different among groups (p = 0.36, Table 1), but no difference in ALT levels was found among groups at 3 and 72 h post-crush (all p > 0.05, Tables 2 and 3).

**Table 1:** The outcome measurement of each group before the crush.

Intervention timing	IPostC modes	ALT	AST	Ca2+	CK	CREA	K	UREA
	Control group	59.54±14.59	121.59±47.52	2.35±0.08	2417.71±1365.40	26.19±8.63	4.84±0.84	5.31±0.72
Immediate (0 min)	A	43.27±2.55*	153.87±16.42	2.40±0.07	2455.43±539.08	20.58±2.30	5.55±0.76	5.91±1.10
	B	41.37±6.33*	128.85±66.85	2.31±0.12	1689.50±953.73	24.25±4.88	5.24±1.05	5.85±0.75
	C	44.75±4.54*	184.18±44.07	2.36±0.16	2079.15±376.51	20.73±3.81	4.96±1.19	5.71±1.01
5 min post-crush	A	43.72±5.74*	132.70±57.40	2.33±0.12	1574.43±763.92	25.70±5.64	5.18±1.24	6.63±1.25
	B	48.10±12.00	132.08±39.80	2.29±0.08	1565.38±395.71	21.83±3.40	5.30±0.70	5.29±0.92
	C	43.92±7.45*	142.62±50.24	2.29±0.10	1853.73±622.02	23.82±5.44	4.97±0.93	5.90±0.97
10 min post-crush	A	49.60±6.74	183.47±38.64	2.23±0.08	2097.65±781.88	22.30±4.18	5.07±0.73	5.38±0.74
	B	44.88±7.77*	166.55±29.73	2.28±0.11	2350.27±703.46	21.77±5.54	5.23±0.77	5.63±0.51
	C	50.23±13.79	184.95±34.85	2.25±0.12	2510.45±822.69	23.93±4.63	5.16±0.53	5.42±0.35
Overall significance, p		0.036	0.068	0.206	0.253	0.535	0.965	0.241

* P < 0.05 compared to the control group; IPostC: ischemic postconditioning; ALT: alanine aminotransferase; AST: aspartate aminotransferase; Ca^2+^: calcium ion concentration; CK: creatine kinase: CREA: creatinine; K: potassium.

**Table 2:** The outcome measurement of each group 3 h post-crush.

Intervention timing	IPost Cmodes	ALT	AST	Ca2+	CK	CREA	K	UREA
	Control group	115.79±98.09	355.99±214.70	2.02±0.25	8213.63±1509.28	81.53±17.22	8.00±1.23	8.83±1.54
Immediate (0 min)	A	77.28±19.30	266.62±101.40	2.24±0.19	6204.97±2233.81	52.12±15.27	5.66±1.12*	13.54±2.36
	B	67.45±27.61	242.92±100.22	2.05±0.28	6021.13±2065.00	55.32±10.07	4.77±2.07*	12.02±2.43
	C	70.10±33.69	228.27±164.44	2.13±0.12	7132.68±1736.50	46.18±17.68*	4.34±1.50*	10.04±3.33
5 min post-crush	A	77.00±27.75	295.65±171.73	2.24±0.25	6163.87±2309.09	69.85±21.06	4.52±1.22*	11.80±3.35
	B	101.38±72.91	327.18±204.27	2.23±0.09	4790.92±2088.95*	52.98±22.20	5.86±1.25	12.37±4.17
	C	103.10±54.08	441.47±229.19	2.10±0.29	5737.92±2118.62	42.35±16.21*	5.08±1.16*	13.17±4.97
10 min post-crush	A	62.18±30.87	165.40±123.29	2.19±0.15	5938.25±2159.37	54.23±26.95	5.81±1.88	10.55±3.47
	B	104.33±54.53	424.40±270.19	2.24±0.06	5415.15±2132.90	64.13±27.04	5.61±1.51*	14.25±1.10*
	C	115.22±57.87	412.12±261.60	2.19±0.13	5778.43±3153.98	54.85±20.82	7.54±1.29	12.36±2.31
Overall significance, p		0.543	0.225	0.356	0.303	0.041	<0.001	0.082

* P < 0.05 compared to the control group; IPostC: ischemic postconditioning; ALT: alanine aminotransferase; AST: aspartate aminotransferase; Ca2+: calcium ion concentration; CK: creatine kinase: CREA: creatinine; K: potassium.

**Table 3:** The outcome measurement of each group 72 h post-crush.

Intervention timing	IPost Cmodes	ALT	AST	Ca2+	CK	CREA	K	UREA
	Control group	42.09±11.91	104.94±47.66	2.76±0.17	4276.71±1766.13	46.33±9.62	6.81±1.34	6.21±0.54
Immediate (0 min)	A	67.63±69.39	251.13±348.34	2.48±0.14*	1056.67±1607.82*	25.55±3.20*	5.14±0.42*	7.18±1.76
	B	76.03±48.71	348.78±307.86	2.39±0.11*	891.70±562.75*	27.33±5.80	5.50±1.05	6.32±1.29
	C	63.83±39.27	244.22±189.60	2.46±0.16*	844.65±613.67*	25.62±9.38*	5.34±1.38*	6.52±1.27
5 min post-crush	A	132.27±113.42	567.30±553.41	2.49±0.07*	827.73±633.84*	22.67±6.25*	5.31±1.10*	7.07±2.20
	B	65.42±27.34	207.38±87.18	2.48±0.13*	625.98±571.32*	24.92±6.85*	5.29±0.80*	6.99±1.71
	C	96.10±68.00	338.82±205.23	2.45±0.12*	898.73±890.86*	24.88±5.83*	4.72±0.77*	8.76±4.01
10 min post-crush	A	54.88±11.75	125.17±58.58	2.46±0.13*	730.22±638.44*	44.08±31.64	5.09±0.55*	7.03±2.70
	B	111.68±107.85	443.27±447.87	2.86±0.36	850.83±841.16*	22.12±3.70*	5.31±0.51*	6.34±0.79
	C	81.73±88.69	401.45±518.96	2.29±0.09*	714.40±508.08*	31.15±12.18	5.07±0.73*	7.19±3.35
Overall significance, P		0.447	0.301	<0.001	<0.001	0.004	0.026	0.717

* P < 0.05 compared to the control group; IPostC: ischemic postconditioning; ALT: alanine aminotransferase; AST: aspartate aminotransferase; Ca^2+^: calcium ion concentration; CK: creatine kinase: CREA: creatinine; K: potassium.

At 3 h post-crush, the CK level of the 5B (5 min post-crush, mode B of IPostC) group, the CREA levels of the 0C (0 min post-crush, mode C of IPostC) and 5C (5 min post-crush, mode C of IPostC) groups, and the K^+^ levels of several groups were all significantly lower than the corresponding control group (all p < 0.05, Table 2). Nevertheless, only the 10B group had a significantly higher UREA level than the control group (p < 0.05, Table 2).

At 72 h post-crush, the levels of Ca^2+^, CK, CREA, and K^+^ of most groups were significantly lower than the control group ones (all p < 0.05, Table 3).

### Comparison of changes in seven indexes among the 15 groups

**Figure 4:**
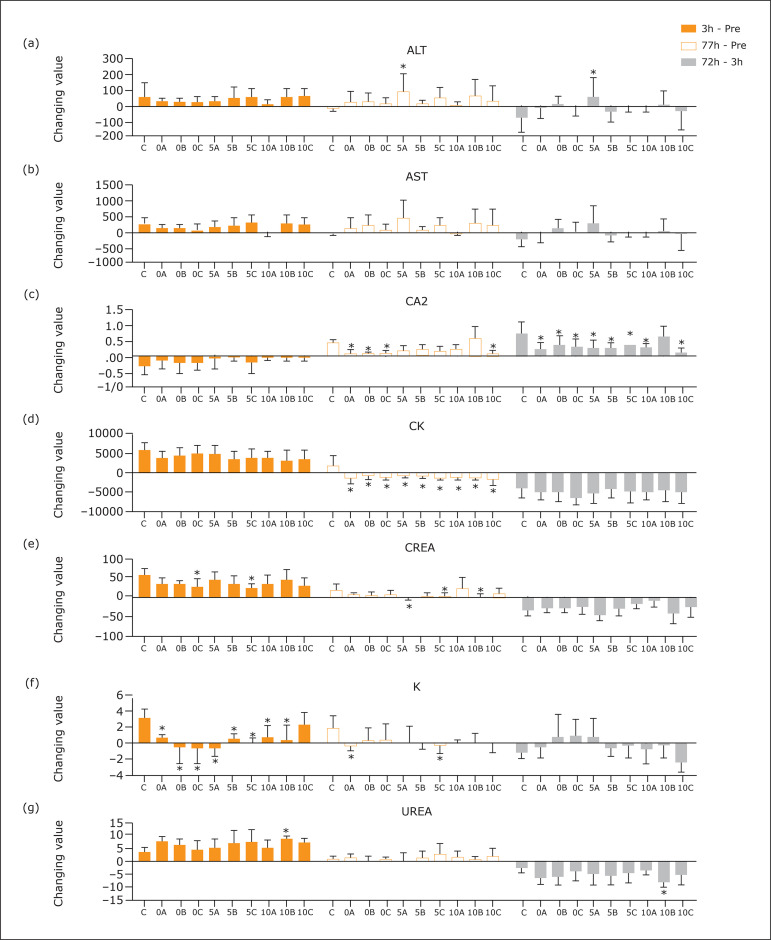
The changing value among different time-points, including 3h – Pre, 72h – Pre, and 72h – 3h of seven indexes: ALT (**a**), AST (**b**), Ca^2+^ (**c**), CK (**d**), CREA (**e**), K (**f**), and UREA (**g**). The initial number 0, 5, and 10 of group name stand for immediate (0 min), 5 min post-crush, and 10 min post-crush of IPost intervention post-crush; the letter A, B, and C of group name stand for different IPostC modes; c stands for the control group. *, P<0.05 compared to the control group.

To further evaluate the efficacy of IPostC interventions, the changes in seven indexes (3 h post-crush – pre-crush, 72 h post-crush – pre-crush, and 72 h post-crush – 3 h post-crush) were compared among the 15 groups. As shown in Fig. 4, the 5A group had significantly higher increasing ALT levels than the control group in both 72 h – pre-crush and 72 h – 3 h (both P<0.05). In the Ca2+ levels, significantly lower changing levels (compared to the control group) were observed in the 0A, 0B, 0C, 10C (72 h – pre-crush) groups and in the 0A, 0B, 0C, 5A, 5B, 5C, 10A, 10 C (72 h – 3 h) groups.

In the CK index, all the nine groups had significantly lower decreasing levels than the control group between 72 h post-crush and pre-crush. In the CREA index, significantly lower decreasing levels were observed in the 0C and 5C (3 h – pre-crush) groups and the 5A, 5C, and 10B (72 h – pre-crush) groups. As for the K^+^ level, significantly lower changing levels compared to the control group were observed in the 0A, 0B, 0C, 5A, 5B, 5C, 10A and 10B (3h – pre-crush) groups, and the 0A and 5C (72h – pre-crush) groups. The 10B group had a significantly higher increasing level of urea at 3 h – pre-crush and a significantly decreasing level of urea at 72 h – pre-crush.

## Discussion

Establishing an animal model of crush injury/syndrome is important for studying the mechanism and treatment of crush syndrome. In 2005, Akimau *et al*.^22^ have established a rat model of crush injury and found that the systemic syndrome of crush injury is closely related to the area of compression. However, their model did not consider the impact of compression weight, time, and area on the severity of local and systemic crush injury. Our previous study found that the increases in compression time, weight, and area can increase the severity of local crush injury and systemic problems in rats, and compression on unilateral hindlimb, compression weight ≥3 kg, compression time ≥ 6 h can cause typical crush syndrome in rats^21^. Therefore, in this study, we used the following conditions for establishing the rat model of crush syndrome: unilateral hindlimb, 3 kg of compression weight, 6 h of compression time.

In this study, the rat’s hindlimb was swollen after 6 h of compression (7.75 ± 0.46 *vs*. 8.46 ± 0.54 mm, p < 0.001), indicating that the compression condition caused local damage to the rat’s limb^23^. The pathological findings also showed local edema and necrosis of the compressed muscle tissue. Crush injury-induced skeletal muscle damage can lead to release of toxic substances, including K, CK, and inflammatory and necrotic molecules into the bloodstream^24^, leading the failure of important organs, such as heart and kidney. *It has been shown that* 13 to 60% of *rhabdomyolysis patients develop acute kidney injury* 25 ^–^ 27.

As a result, serum levels of K^+^, CREA, and CK could be used as markers for assessing the severity of crush syndrome28. In this study, serum levels of CK at 3 h post-crush had become 2.3 to 3.9 times among all 10 groups as compared with that before crush, suggesting the crush model was successfully established. At 3 h post-crush, there were only a few significant differences in all parameters between the nine intervention groups and the corresponding control group. At 72 h, serum CK level was reduced to 1.17 time of that before crush in the control group. However, the CK level was reduced to 0.28-0.53 time of the one before crush in all intervention groups. Likewise, the CREA level was elevated to 3.11 times in the control group at 3 h post-crush and reduced to 1.77 time at 72 h post-crush.

Nevertheless, at 72 h post-crush, the CREA level in all intervention groups ranged from 0.88 to 1.98 time (< 1.3 time in six group). As for serum K, the K level was elevated to 1.65 and 1.41 time in the control group at3 h and 72 h post-crush, respectively. However, the K levels were maintained 0.87 to 1.15 time at 3 h post-crush among eight of nine intervention groups and 0.88 to 1.24 time at 72 h post-crush among seven of nine intervention groups.

These results suggest that the IPostC intervention can protect the crushed rats from crush-induced elevation of serum CK, CREA and K levels, which is in line with previous findings^13^^–^ 17. After the compression was removed, the IPostC intervention allowed the toxic substances released from the damaged skeletal muscles into the circulation in batches, avoiding a large number of toxic substances, simultaneously entering the bloodstream and causing irreversible damage.

At present, there is no consensus on the ideal postconditioning protocol. To determine the optimal IPostC intervention protocol, we designed three timing of IPostC intervention (0 min, 5 min, and 10 min post-crush) and three modes of IPostC. Although the protective effect of IPostC intervention could be observed in all the intervention groups, it seemed that the IPostC intervention at 10 min post-crush groups had a less protective effect in terms of the CREA level at 72 h post-crush. In addition, the H,E staining also suggested that the 10A, 10B and 10C groups had more severe damage of muscle tissue and inflammatory cells infiltration. This observation is consistent with previous studies that postconditioning interventions should be started immediately at the time of initial reperfusion^29^^–^ 31. Considering the results of H,E and blood biochemical analysis, the 0B, 0C, 5A and 5C groups seemed to provide better protection.

There are still some limitations to this study. The compression weight used here was 3 kg, which can prevent the rats from retracting the compressed limbs. However, in earthquakes and accidents, the compression weight maybe hundreds of times the weight of the injured person. Then, the impact of greater compression weight also needs further investigation. In addition, since this study has many groups, the sample size of each group is too small. In the future, a well-designed study with a large sample size should be conducted to verify the findings of this investigation.

## Conclusions

In summary, our IPostC intervention protocols can effectively protect the rats from crush-induced elevation of serum CK, CREA, and K+ levels. The timing of IPostC intervention should be as early as possible, to ensure the protective effect.
